# A cross-sectional study on the use and misuse of trypanocides in selected pastoral and agropastoral areas of eastern and northeastern Tanzania

**DOI:** 10.1186/s13071-017-2544-3

**Published:** 2017-12-15

**Authors:** Anna F. Ngumbi, Richard S. Silayo

**Affiliations:** 1 Livestock Training Agency, PO Box 603, Morogoro, Tanzania; 2 Department of Microbiology, Parasitology and Biotechnology, Sokoine University of Agriculture, PO Box 3019, Chuo Kikuu, Morogoro, Tanzania

**Keywords:** Trypanocides, Insecticides, Trypanosomosis control, Tsetse, Veterinary service delivery, Drug misuse

## Abstract

**Background:**

Tsetse-borne African animal trypanosomosis (AAT) greatly influences livestock distribution and significantly slows livestock productivity in sub-Saharan Africa. While a number of control methods targeting the vector tsetse are in field application, treatment with the few available trypanocides continues to be the most widely applied control method. Unfortunately, improper and frequent use of these few available drugs, accelerated by poor veterinary service delivery, promotes trypanosome drug resistance, the magnitude of which has not been delineated. In the present study, current practices on trypanocide application for the control of bovine trypanosomosis in the field in Tanzania were studied with a view to providing policy advice on the safe and sustainable use of trypanocides.

**Methods:**

A cross-sectional study was conducted using a semi-structured questionnaire administered to a total of 200 randomly selected livestock keepers in selected pastoral and agropastoral areas within three districts in eastern and north eastern Tanzania.

**Results:**

In total, 50% of respondents in all three study districts had primary level education; over 40% had informal education and only 5% with university education (all from one district, Pangani). Age-wise, most respondents were aged 30–59 years with exception of Korogwe where 35% were aged 20–29 years. Over 95% of respondents had knowledge on tsetse as a vector of trypanosomosis and correctly identified tsetse in provided pictures. Furthermore, 98.7% of the respondents applied pyrethroids for vector control. Regarding parasite control practices, this study revealed a high degree of variation in trypanocides usage and the intervals of their application. Whereas only 20% of respondents use chemoprophylaxis for trypanosomosis control, the majority (69–95%) wrongly use diminazene aceturate thinking it is prophylactic, while only 5–30% used the prophylactic drug isometamidium chloride. Most of the respondents (95% in Korogwe, 60% in Pangani and 93.1% in Mvomero) administered the drugs on their own. Improper trypanocides administration was high as respondents in Korogwe (75%) and Mvomero (72%) administered the drugs intravenously with a view to achieving faster drug effect contrary to manufacturers’ recommendations, while 40% of respondents from Pangani used both intravenous and intramuscular routes. Additionally, respondents did not observe the recommended withdrawal periods for the drugs.

**Conclusions:**

This study revealed a high level of trypanocides misuse which poses a high risk of trypanosome drug resistance development as well as risks to human health from drug residues in consumed animal products. This calls for improvements in veterinary service delivery in pastoral and agropastoral areas of Tanzania to counteract the misuse of chemotherapeutics.

**Electronic supplementary material:**

The online version of this article (10.1186/s13071-017-2544-3) contains supplementary material, which is available to authorized users.

## Background

Tsetse-borne African animal trypanosomosis (AAT) causes significant economic losses as it lowers livestock productivity [1, 2] and highly influences livestock distribution in Africa [3, 4]. In endemic areas of Tanzania, control of the disease relies on vector control as well as parasite (trypanosome) control [5, 6].

Parasite control by chemotherapy and chemoprophylaxis predominates as a method of choice in controlling AAT [7] as it is easier than controlling the vector, especially in communities where livestock production involves transhumance in search of water and pasture [8]. Trypanocides are probably the most commonly used veterinary drugs in sub-Saharan Africa [9]). It is estimated that 35 million doses of trypanocides are used annually in Africa where about 50–70 million animals are at risk of getting trypanosomosis [2, 9, 10]. However, the use of drugs to treat and control trypanosomosis is fraught with a number of problems including paucity of available drugs, trypanosome drug resistance development [11, 12] and lack of industry interest in discovery and development of new trypanocidal drugs for field use.

Despite animal trypanosomosis being controlled principally by trypanocides, farmers depend on only three compounds, namely, homidium chloride/bromide, diminazene aceturate and isometamidium chloride, the last of which was first released for field use more than 55 years ago [7, 13]. Prospects for new drugs being made available for field application against trypanosomoses are few on account of the African market for the drugs being unattractive to the pharmaceutical industry [14].

As a measure to ensure the available drugs remain useful for a long time, there is need for judicious use of the currently available drugs to slow down the speed of trypanosome drug resistance development and there has been a call for close monitoring of trypanocide usage under field conditions [9].

The way trypanocidal drugs are being used in the field in Tanzania can be related to circumstances which began in 1984 when the government devolved veterinary service delivery into private hands. This has led to near-zero availability of such service in pastoral areas, particularly in areas where transhumance for pasture and water prevails, as a consequence of such areas being commercially and logistically unattractive. As a result, untrained pastoralists are delivering the veterinary service themselves adding unnecessary cost to livestock production from overuse/misuse along with the risk of drug resistance development, danger to health of those handling the drugs, and danger to those consuming the animal products.

In Tanzania, veterinary drugs including trypanocides were strictly controlled by the veterinary department [15]. However, recently with the privatization of veterinary services, veterinary pharmaceuticals including trypanocides are freely available. These drugs can currently be purchased directly by farmers from local veterinary shops and, more alarmingly, from livestock markets where they are often displayed under the heat of the sun. This study was conducted to establish the level of use and misuse of trypanocides in selected pastoral communities of Tanzania with a view to providing policy advice in mitigating the problem of trypanosome drug resistance development.

## Methods

### Study area and design

A multistage randomized survey was employed and the districts of Korogwe and Pangani in Tanga region (northeastern Tanzania) and Mvomero in Morogoro region (eastern Tanzania) were selected. Village information was obtained from the district veterinary officers (DVOs) and selection of villages for survey was purposive based on information on the presence of pastoral communities, the reported presence of tsetse/ trypanosomosis and the reported use of trypanocides. In Korogwe district (5°0′S, 38°25′E; elevation 1093 m above mean sea level (amsl)), surveys were carried out in three villages, namely, Mkalamo, Mgambo and Changalikwa while in Pangani (5°30′S, 38°49′E; elevation 69 m amsl), the surveys were carried out in two villages, namely, Masaika and Mivumoni. These two villages differed in terms of average herd size which was 300 and 20–100 for Mivumoni and Masaika respectively. In Mvomero district (6°21′S, 38°27′E; elevation 160 m amsl), the villages selected for study were Msongozi, Mangae, Mela, Wami luhindo and Kambala.

### Questionnaire administration

The study involved the administration of a questionnaire to heads (or representatives) of households randomly selected from a list of those owning cattle as provided by ten cell leaders. A total of 200 cattle owners/ trypanocide users participated in the questionnaire study. The questionnaire was semi-structured containing questions to assess knowledge as well as practice. Questions sought to assess (i) knowledge on clinical signs of trypanosomosis; (ii) identification of tsetse flies from presented pictures; (iii) the presence of flies of that kind in their areas and determine types of control measures applied; (iv) how controls were carried out; (v) knowledge on the use of trypanocidal drugs as control measures for trypanosomosis; (vi) preference of trypanocidal drugs; (vii) routes and frequency of drug administration; (viii) drug dilution; and (ix) observation of withdrawal period (see Additional file 1). The focus was on isometamidium use as a prophylactic agent, though questions were also directed on the use of the curative drug diminazene aceturate to a lesser extent. Furthermore, questions were posed to explore their knowledge and attitudes on the application of pyrethroid insecticides.

### Data analysis

Data obtained were entered into excel data sheets and descriptive statistics were obtained using Epi-info software programme, version 7.

## Results

### Respondent demographic characteristics

**Fig. 1:**
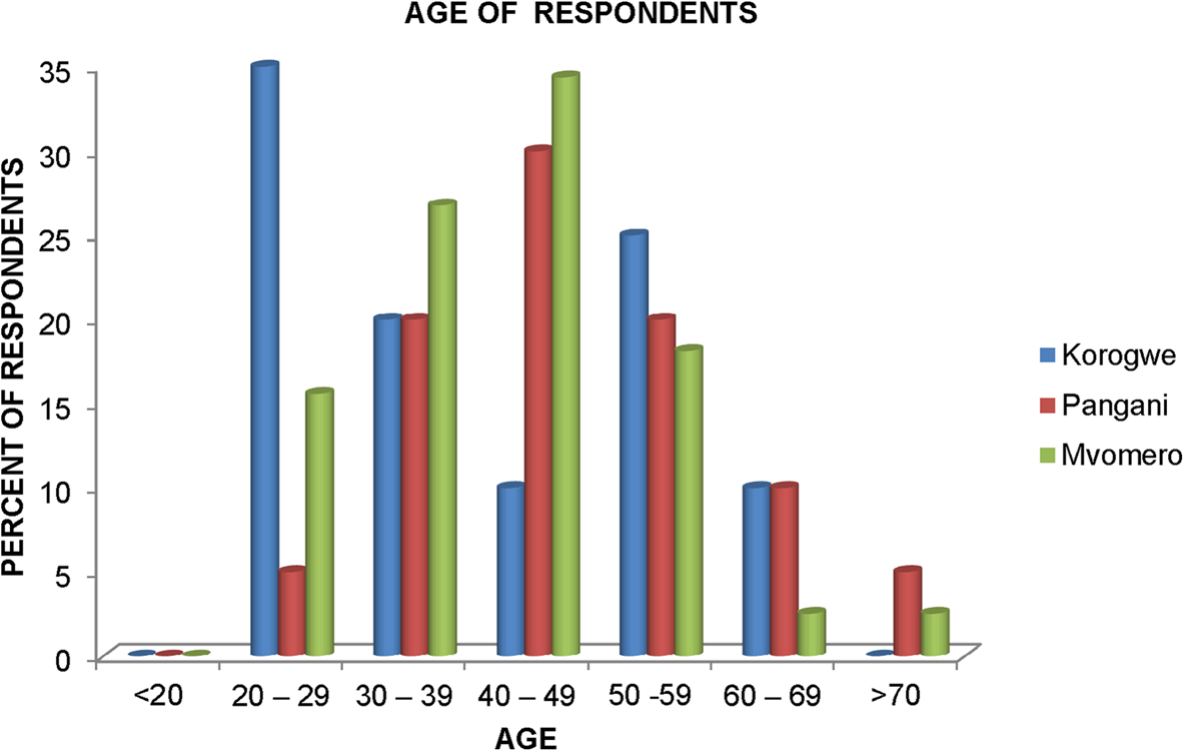
Age distribution of respondents interviewed in the three study sites in Tanzania

A total of 200 cattle owners were interviewed, of which 131 (65.5%) were males and 69 (34.5%) were females. Most of the respondents were in the age group 20–59 years in all three study sites, with the majority being 40–49 years old in Mvomero and Pangani, and an exceptionally high number of respondents in the age group 20–29 years in Korogwe (Fig. 1). The vast majority (75, 80 and 71%, respectively) of respondents in Korogwe, Pangani and Mvomero were heads of households.

**Fig. 2:**
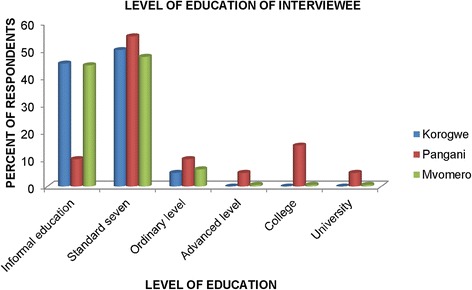
Interviewee level of formal education in the study areas in Tanzania

Generally, most of the respondents had informal or primary level education. Korogwe and Mvomero districts had relatively higher percentages of respondents with informal education than Pangani district where more respondents had formal education, including 5% advanced level secondary education, 15% college and 5% university education (Fig. 2).

### Respondents’ knowledge and practice on tsetse, tsetse control methods and trypanosomosis

Respondent awareness on presence of the disease was high in Pangani and Mvomero districts (75% and 74%, respectively), but was low in Korogwe (30%) (Table 1). Tsetse flies are known to most of the farmers as it was shown that 95% to100% of the respondents in all three districts knew and have observed them in the field. The results indicated that 99% of respondents in Mvomero and 100% respondents in both Korogwe and Pangani apply pyrethroids on their animals as a control measure against ticks and tsetse flies. Ninety five percent of respondents in both Korogwe and Pangani, and 100% in Mvomero, use insecticides without additional bush clearing or target panel application (Table 1).

**Table 1:** Responses on trypanosomosis and tsetse control in different study districts

Question	Response	Korogwe (%)^a^	Pangani (%)^a^	Mvomero (%)^a^
Presence of trypanosomosis	Yes	30	75	74
	No	70	25	26
Presence of tsetse flies	Yes	95	100	98
	No	5	0	2
Tsetse flies control methods	Insecticide application	95	95	100
	Bush clearing	0	0	0
	Target panel	0	0	0
	Insecticide applicationwith either of the other two^b^	5	5	0
Pyrethroid application	Yes	100	100	99
	No	0	0	1

^a^Percentage distribution of interviewees’ responses in Korogwe, Pangani and Mvomero for each question asked

^b^Insecticides application with either bush clearing or target panel

Tsetse control as a method for trypanosomosis control was mentioned by 45, 7 and 0% of respondents from Pangani, Mvomero and Korogwe, respectively (Table 2). Chemoprophylaxis for trypanosomosis control was a method practiced by 20, 10 and 11% of respondents from Korogwe, Pangani and Mvomero districts, respectively. On the other hand, 60 and 43% of respondents from Korogwe and Mvomero, respectively indicated they use chemotherapy as control method in contrast to Pangani where none of the respondents indicated using chemotherapy. The remaining 20, 45 and 39% respondents from Korogwe, Pangani and Mvomero, respectively indicated that they combine tsetse control with either chemoprophylaxis or chemotherapy interchangeably. Furthermore, 5, 45 and 33% respondents of Korogwe, Pangani and Mvomero districts, respectively use chemoprophylaxis as a routine way of controlling trypanosomosis.

**Table 2:** Trypanosomosis control approaches opted by respondents in Korongwe, Pangani and Mvomero districts

Question	Response	Korogwe (%)^a^	Pangani (%)^a^	Mvomero (%)^a^
Trypanosomosis control method	Tsetse control	0	45	7
	Chemoprophylaxis	20	10	11
	Chemotherapy	60	0	43
	Slaughter	0	0	1
	Tsetse control with either of the two^**b**^	20	45	39
Routine chemoprophylaxis	Yes	5	45	33
	No	95	55	67
Drug used for prophylaxis	ISMM	5	45	31
	DA	95	30	69

^a^Percentage distribution of interviewees’ responses in Korogwe, Pangani and Mvomero for each question asked

^b^Tsetse control with either chemoprophylaxis or chemotherapy

*Abbreviations*: ISMM, isometamidium chloride; DA, diminazene aceturate

Very few (5 and 31%) respondents in Korogwe and Mvomero, respectively indicated that they use isometamidium for prophylaxis while the remaining 95 and 69% used diminazene aceturate. The situation is different in Pangani where 45% of respondents administered isometamidium for prophylaxis and 30% used diminazene aceturate.

**Fig. 3:**
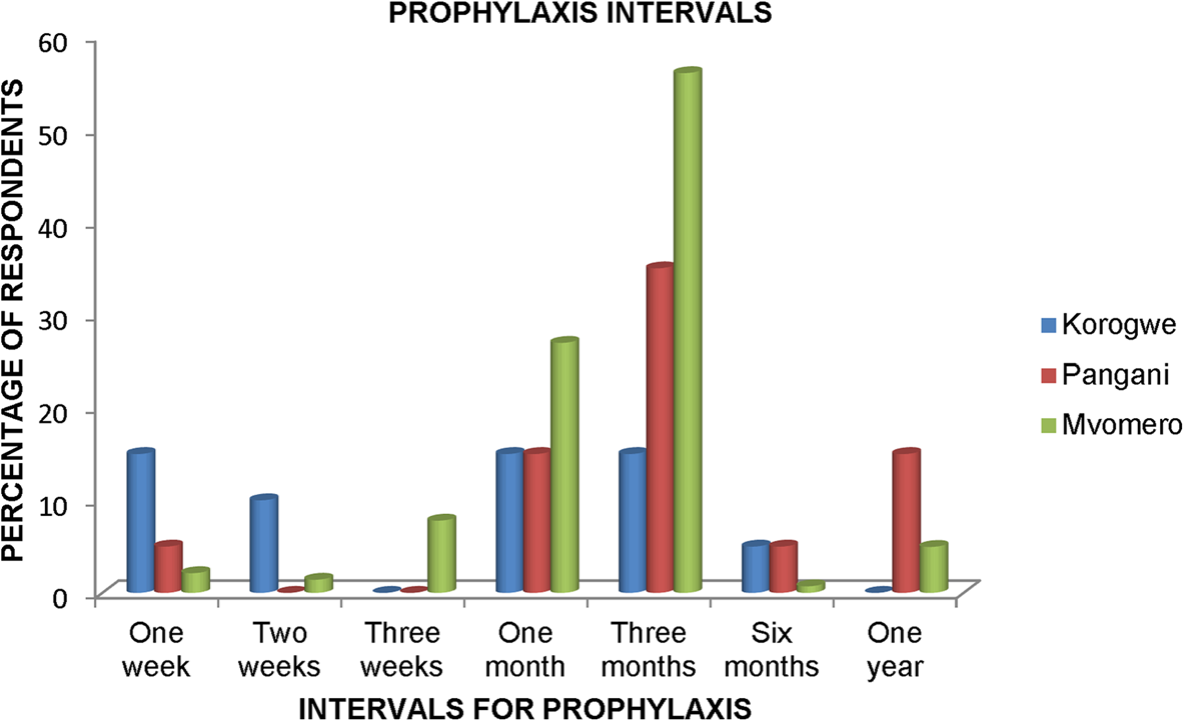
Responses on intervals of chemoprophylaxis administration

Responses on intervals of isometamidium administration showed considerable variation. More farmers, especially in Mvomero (56%), indicated that they provide prophylaxis after every 3 months, but there was a wide range from 1 week to as much as 1 year. The extremes were from 15% of respondents from Korogwe applying isometamidium once a week and 15% in Pangani applying isometamidium once a year (Fig. 3).

### Availability of drugs and knowledge on drug indications and adherence to the indications

All Pangani respondents indicated that they obtained drugs from veterinary pharmacies in contrast to those from Korogwe and Mvomero, of whom 35 and 14%, respectively indicated they obtain their animal drugs from veterinary pharmacies. Thirty five and eighty one percent of respondents from Korogwe and Mvomero, respectively indicated they obtained the drugs from both veterinary pharmacies and livestock markets. Those indicating they obtained the animal drugs only from livestock auction markets were 30 and 3.1% from Korogwe and Mvomero, respectively.

Knowledge on withdrawal period was high in Pangani (85%) followed by Mvomero (70%) and lastly in Korogwe (60%). However, despite a good knowledge on withdrawal period 32, 72 and 80%, of respondents from Pangani, Mvomero and Korogwe, respectively, indicated they were consuming products from treated animals at periods less than the withdrawal periods. Reasons given by those consuming products from treated animals were a lack of awareness of the associated risks as mentioned by 100% of respondents from Korogwe, 25% from Pangani and 85% in Mvomero, while 75 and 15% of respondents from Pangani and Mvomero respectively, indicated that they consumed as a way of avoiding economic loss from discarding the product (Table 3).

**Table 3:** Sources of drugs and knowledge on indications and adherence to the indications

Question	Response	Korogwe (%)^a^	Pangani (%)^a^	Mvomero (%)^a^
Drug availability^**b**^	Veterinary pharmacies	35	100	14
	Veterinary professional	0	0	1
	Livestock auction market	30	0	3
	Veterinary pharmacies & livestock markets	35	0	81
Withdrawal period if known^**c**^	Yes	60	85	70
	No	40	15	30
Products from treated animal^**d**^	Thrown away	15	47	26
	Used for human consumption	80	32	72
	Consumed by other animals	5	21	2
Reason for not observing withdrawal period^**e**^	Unaware of risks to be encountered	100	25	85
	Avoiding economic loss	0	75	15

^a^Percentage distribution of interviewees’ responses in Korogwe, Pangani and Mvomero for each question asked

^b^What was the source of the drugs

^c^Respondents knowledge on drugs withdrawal period

^d^How the products from treated animals were handled

^e^Any reason for not observing withdrawal period

**Fig. 4:**
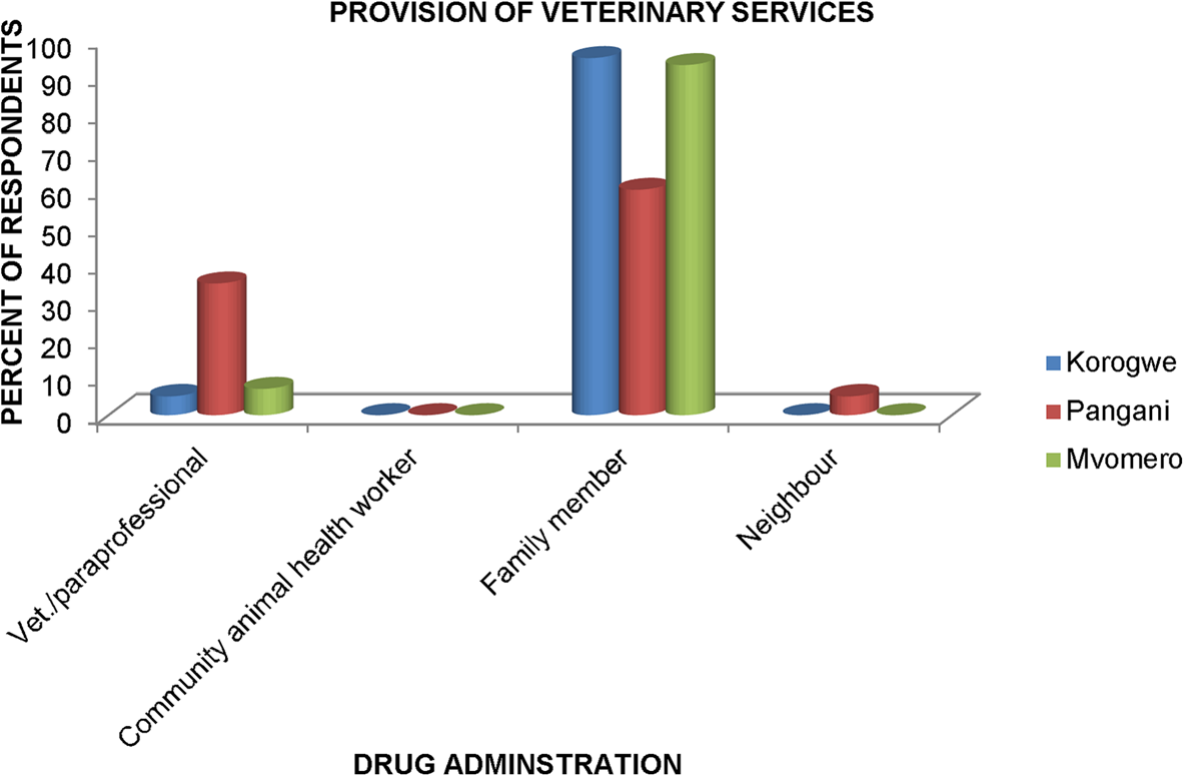
Responses on who administers drugs to the animals

In all the three districts, drug administration as indicated by 95, 60 and 93% of respondents in Korogwe, Pangani and Mvomero, respectively, was carried out by family members (Fig. 4). Pangani district was the only district where a considerable proportion (35%) of respondents indicated getting veterinary services from veterinary professionals, paraprofessionals and assistant paraprofessionals (Fig. 4).

**Fig. 5:**
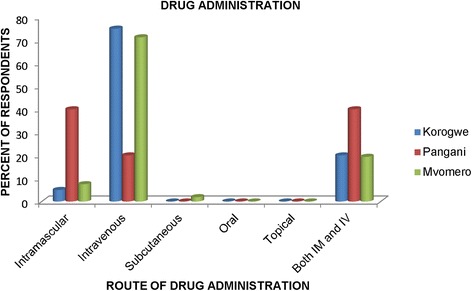
Responses on the route of drug administration

Unprofessional handling of drugs was observed when respondents answered a question on the route of trypanocidal drugs administration whereby most (75 and 72%, respectively) of respondents in Korogwe and Mvomero indicated that they prefer to use an intravenous (IV) route to administer the drugs rather than the recommended deep intramuscular (IM) route. The situation was better in Pangani where 40% indicated they use the recommended IM route and a similar proportion (40%) indicated they use IV and IM routes interchangeably (Fig. 5) regardless of the risk encountered when using intravenous route. Respondents admitted that they use the IV route because it results in faster drug action.

Generally, respondents gave different remarks as to why they did not predominantly practice control of trypanosomosis using trypanocides, though most of them (with exception of 10% respondents from Mvomero) seemed willing to control the disease using trypanocides. There were farmers who indicated that there was an unavailability of drugs, particularly isometamidium (samorin), and these were 20% from Korogwe, 35% from Pangani and 45% from Mvomero. Lack of knowledge was another factor mentioned by 15, 5 and 20% of respondents in Korogwe, Pangani and Mvomero, respectively. Insufficient veterinary services was a complaint from 45% respondents in Korogwe, 10% of those from Pangani and 18% of those from Mvomero, Financial constraint was not an issue in Korogwe, while this was mentioned by 7% of respondents from Mvomero and a relatively high proportion (35%) of respondents from Pangani (Table 4).

**Table 4:** General comments given by farmers on the use of trypanocides for controlling animal trypanosomosis

General observation	Korogwe (%)^a^	Pangani (%)^a^	Mvomero (%)^a^
Unavailability of drugs	20	35	45
Unwillingness to control	0	0	10
Lack of knowledge	15	5	20
Lack of veterinary services	45	10	18
Financial constraint	0	35	7

^a^Percentage distribution of interviewees’ responses in Korogwe, Pangani and Mvomero for each question asked

## Discussion

This study has indicated that animal trypanosomosis is still perceived as an important animal disease in the study areas, leading to widespread use of the trypanocides, diminazene aceturate and isometamidium chloride. Many respondents in this study acknowledged the presence of trypanosomosis in their areas which concurs with observations by Nonga and Kambarage [5] and the presence of tsetse flies in the history of their areas, especially in villages of Korogwe and Mvomero, similarly reported by Malele. [16]. Despite more recent report [17], which states that the area of Tanzania currently occupied by the tsetse is now only one third, a dramatic decrease from the previous two thirds, tsetse-transmitted animal trypanosomosis is still one of the major health problems affecting livestock in the study areas.

Respondents had varying levels of education with most having a primary level of education. The literacy level in Pangani respondents was proportionally higher than in Korogwe and Mvomero. This also corresponded to the ability of the respondents to understand the diseases and the decision on who treats the animals, which drug to use and where to purchase the drugs. In Pangani district, respondents appeared more knowledgeable on the disease and its control than in the other two districts, Korogwe and Mvomero. The knowledge level of Pangani respondents can be attributed to the existence of two missionary centers for sisters and brothers (Capuchin Franciscan sisters and brothers) but also largely due to the Mivumoni Tsetse and Trypanosomosis research farm; a few respondents were retired workers of this farm, so they have dealt with tsetse and trypanosomiasis before. This knowledge level was comparable to studies previously reported in Tanzania [18] and Kenya [19].

Despite the differences in their levels of formal education, respondents in all districts were knowledgeable on tsetse and trypanosomosis. A high proportion of respondents indicated that they can identify tsetse flies which they were able to do in pictures. Similar knowledge has been reported in and around Serengeti National Park [18, 20]. They have knowledge on the control means and a larger proportion indicated use of insecticide as a choice over bush clearing or target panel. In all study areas, the use of pyrethroid insecticides to simultaneously control tsetse flies and tick-borne diseases was well adopted by respondents, corresponding to similar findings by Magwisha et al. [21] and Muhanguzi et al. [22]. Additional information from the farmers (not included in questionnaire) indicated that the frequency of insecticides application differed among farmers ranging from daily to 2 week intervals. This was influenced by family manpower whereby households with more children applied insecticides more frequently as they were using hand sprayers.

In some cases, livestock farmers may not be aware that they are controlling tsetse through vector control by insecticide application. In Korogwe, for example, there was considerable use of pyrethroids on cattle ostensibly for tick and tick-borne disease control but they were not aware that the acaricide was also very effective as an insecticide against tsetse. It was also at Korogwe that livestock keepers showed ignorance on how trypanosomosis is transmitted; some believed cattle only get an infection when they lick a site where a tsetse has just bitten. These findings correspond to the observation made in Kenya [23], southwestern Ethiopia [24] and Tanzania [20].

Chemoprophylaxis and chemotherapy were mentioned as a means of trypanosomosis control, but chemotherapy (whereby farmers administered trypanocides when the disease was encountered) was being employed more than chemoprophylaxis. However, where chemoprophylaxis was applied, application regime differed among farmers, with a frequency ranging from once per week to once per year. This study revealed that a frequency treatment at one-week intervals was practiced in Korogwe, while a prolonged interval (one-year) was practiced by some respondents in Pangani. A three-month interval (as per manufacturer’s recommendation) was practised in Mvomero district. This variation in prophylactic regimes, especially the annual interval, may promote emergence of the drug resistant trypanosomes as pointed out by others [6, 25–27].

The present study has indicated use of the curative diminazene aceturate in preference to the prophylactic isometamidium chloride for trypanosomosis control. Use of curative trypanocidal drugs, such as diminazene for prophylactic purposes, is regarded as mass treatment in which both infected and non-infected animals are treated. Treatments of uninfected animals results in unnecessary cost to animal production while the treatment of animals which happen to be infected results in bonus immunity against the local stocks of trypanosomes. It can, therefore, be said that use of diminazene in mass treatment in situations where there is possibility of laboratory confirmation of infection is a misuse of the drug. Diminazene aceturate is rapidly cleared from the body reducing its potentiality for use as a prophylactic drug [28]. Misuse of these drugs may be associated with inadequate veterinary service providers in their areas and a tendency of farmers to obtain drugs from informal traders who sell these drugs in livestock auction markets without information on their correct use. A similar trend of farmers acquiring drugs from unqualified service providers was reported by Machila et al. [29] in Busia and Kwale Districts in Kenya. Veterinary service provision is greater in Pangani when compared to Korogwe and Mvomero, and whilst geographic coverage may be a main reason, the level of education of livestock keepers may also have an influence.

This study has also shown that farmers are left with no option but to treat their own animals due to inadequate veterinary service delivery in the study area. This is the case in many countries where provision of veterinary services is insufficient and many small farmers deliver animal health services [30]. As a result of this practice, where treatment is done by farmers themselves and there is limited training of community animal health workers, there is a high degree of misuse of drugs as similarly observed by Geerts et al. [9]. This is as a result of the wrong drug choice (considering the ability of farmers to make a diagnosis is limited since most of them rely on clinical signs), incorrect routes of drug administration, inconsistent administration intervals for prophylaxis and, more importantly, ignored observation of the withdrawal period as a consequence of disregarding (or lack of knowledge) manufacturers’ recommendations. As a result of this haphazard use of drugs, the possibilities of creating resistant strains of trypanosomes are high. This calls for emphasis on training more paraprofessionals and assistant paraprofessionals amongst transhumant pastoralists.

A general observation from this study is that the availability of different approaches for control of trypanosomosis results in livestock keepers rarely seeking veterinary assistance in managing the disease. Despite the small number of veterinarians, most farmers showed a desire to receive more veterinary assistance in controlling other diseases such as viral diseases (FMD) and tick-borne diseases, especially heartwater. This could be due to the fact that today trypanocidal drugs are available at an affordable price when compared to drugs for tick-borne diseases, as also observed by Magwisha et al. [21]. Furthermore, the use of insecticides, particularly pyrethroids, permits cattle and tsetse flies to co-exist.

## Conclusions

The present studies have corroborated a previously reported misuse of trypanocides in Tanzania as a consequence of poor veterinary service delivery in transhumant pastoral areas that are unattractive to private veterinary practice. Experience shows that even private veterinary service delivery faces difficulties in a pastoral setting due to livestock movements and lack of permanent settlements, leading to most of the livestock keepers in such situations left with no choice but to deliver the veterinary service on their own without proper training. Because diagnosis is largely clinical-sign based, treatment is unspecific and adds to animal production costs. Such haphazard use of trypanocides is likely promoting trypanosome resistance to the few drugs still available for field use and moreover due to lack of observation of drug withdrawal periods, resulting in health risks to consumers of the animal product. To overcome these problems we recommend that more studies are carried out on the issue of veterinary service delivery in transhumance pastoral situations, especially where vector-borne parasitic diseases are a concern. Furthermore, the education of farmers on the importance of laboratory-based disease diagnosis, indications of trypanocidal drugs, and consequences of not adhering to those indications both economically and for public health concerns is of importance.

## Additional file

Additional file 1: Questionnaire on current use of trypanocidal drugs. (DOC 223 kb)

## Abbreviations

AAT: African animal trypanosomosis
amsl: Above mean sea level
COSTECH: Commission for Science and Technology
DVO: District veterinary officer
IM: Intramuscular
IV: Intravenous
SUA: Sokoine University of Agriculture

## References

[1] Mersha C, Dulecha A, Basaznew B (2013). Socio-economic assessment of the impacts of trypanosomiasis on cattle in Girja District, southern Oromia region, southern Ethiopia. Acta Parasitol Glob.

[2] Shaw APM (2009). Assessing the economics of animal trypanosomosis in Africa - history and current perspectives. Onderstepoort J Vet Res.

[3] Swallow BM (2000). Impacts of trypanosomiasis on African agriculture. PAAT Sci Tech Series.

[4] Jahnke HE, Tacher G, Keil P, Rojat D (1988). Livestock production in tropical Africa, with special reference to the tsetse-afferent zone. In the livestock production in tsetse affected areas of Africa. The African trypanotolerant livestock network.

[5] Nonga HE, Kambarage DM (2009). Prevalence of bovine trypanosomiasis in Morogoro, Tanzania. Pakistan J Nutr.

[6] Silayo RS, Kimbita EN, Mutayoba BM, Maselle RM (1996). Use and abuse of acaricides and trypanocides in the field. Preliminary findings from Morogoro. Tanzania Vet J.

[7] Holmes PH, Eisler MC, Geerts S, Maudlin I, Holmes PH, Miles MA (2004). Current chemotherapy of animal trypanosomiasis. The trypanosomiases.

[8] Jordan AM (1994). Arguments for and against considering trypanosomiasis as different from other animal diseases. In a systematic approach to tsetse and trypanosomiasis control.

[9] Geerts S, Holmes PH, Diall O, Eisler M (2001). African bovine trypanosomiasis: the problem of drug resistance. Trends Parasitol.

[10] Kristjanson PM, Swallow BM, Rowlands GJ, Kruska RL, De leeuw PN (1999). Measuring the costs of African animal trypanosomosis, the potential benefits of control and returns to research. Agric Syst.

[11] Delespaux V, Geysen D, Van De Bossche P, Geerts S (2008). Molecular tools for the rapid detection of drug resistance in animal trypanosomes. Trends Parasitol.

[12] Geerts S, Holmes PH (1998). Drug management and parasite resistance in bovine trypanosomiasis in Africa.

[13] Achenef M, Bekele B (2013). Drugs and drug resistance in African animal trypanosomosis: a review. Europ J Appl Sci.

[14] Shiferaw S, Muktar Y, Dinaol B (2015). A review on trypanocidal drug resistance in Ethiopia. J Parasitol Vect Biol.

[15] National Livestock Policy (2006). Ministry of livestock development. The United Republic of Tanzania.

[16] Malele II. Fifty years of tsetse control in Tanzania: challenges and prospects for the future. Tanzan J Health Res. 2011;13(5 Suppl. 1):399–406. 10.4314/thrb.v13i5.9 26591994

[17] Daffa J, Byamungu M, Nsengwa G, Mwambene E, Mleche M (2013). Tsetse distribution in Tanzania: 2012 status. Tanzania Vet J.

[18] Kinung’hi SM, Malele II, Kibona SN, Matemba LE, Sahani JK, Kishamawe C, Mlengeya TDK (2006). Knowledge, attitudes and practices on tsetse and sleeping sickness among communities living in and around Serengeti National Park, Tanzania. Tanzania Health Res Bull.

[19] Machila N, Emongor R, Shaw AP, Welburn SC, Mc Dermott J, Maudlin I, Eisler MC (2007). A community education intervention to improve bovine trypanosomiasis knowledge and appropriate use of trypanocidal drugs on smallholder farms in Kenya. Agric Syst.

[20] Byamungu M, Nkwengulila G, Matembo S (2016). Evaluation of knowledge, attitude and practices of agropastoralists on tsetse fly (*Glossina* sp.) in western Serengeti, Tanzania. J Vet Med Anim Health.

[21] Magwisha HB, Malele II, Nyingilili HS, Mamiro KA, Lyaruu EA, Kapange LA (2013). Knowledge, attitude and control practices of tsetse flies and trypamosomiasis among agro-pastoralists in Rufiji Valley, Tanzania. J Commonwealth Vet Ass.

[22] Muhanguzi D, Okello OW, Kabasa JD, Waiswa C, Welburn SC, Shaw APM (2015). Cost analysis of options for management of African animal trypanosomiasis using interventions targeted at cattle in Tororo District, South-eastern Uganda. Parasit Vectors.

[23] Ohaga SO, Kokwaro ED, Ndiege IO, Hassanali A, Sain RK (2007). Livestock farmers perception and epidemiology of bovine trypanosomosis in Kwale District, Kenya. Prev Vet Med.

[24] Seyoum Z, Terefe G, Ashenafi H (2013). Farmers’ perception of impacts of bovine trypanosomosis and tsetse fly in selected districts in Baro-Akobo and Gojeb river basins, southwestern Ethiopia. Vet Res.

[25] Roderick S, Stevenson P, Mwendia C, Okech G (2000). The use of trypanocides and antibiotics by Maasai pastoralists. Trop Anim Health Prod.

[26] Van den Bossche P, Doran M, Connor RJ (2000). An analysis of trypanocidal drug in the eastern province of Zambia. Acta Trop.

[27] Magona JW, Walubengo J, Olaho-Mukani W (2004). Knowledge and attitudes of cattle owners regarding trypanosomiasis control in tsetse-infested areas of Uganda. J South African Vet Ass.

[28] Delespaux V, de Koning HP (2007). Drugs and drug resistance in African trypanosomiasis. Drug Resist Update.

[29] Machila N, Eisler MC, Wanyangu SW, McDermott JJ, Welburn SC, Maudlin I (2003). Cattle owners perceptions of African bovine trypanosomiasis and its control in Busia and Kwale districts of Kenya. Acta Trop.

[30] Grace D, Jost C, Macgregor-Skinner G, Mariner JC. Participation of small farmers in animal health programmes. Tech. Item no. 1. In: OIE, Final report of the 76th General Session, France-Paris, 25–30 May; 2008. p. 19–34.

