# Supplementary material for: A cross-sectional study on the use and misuse of trypanocides in selected pastoral and agropastoral areas of eastern and northeastern Tanzania
Source: Parasit Vectors. 2017 Dec 15;10:607. doi: 10.1186/s13071-017-2544-3 (PMC5731095; doi:10.1186/s13071-017-2544-3)
Supplement: Additional file 1: — Questionnaire on current use of trypanocidal drugs. (DOC 223 kb) [file 13071_2017_2544_MOESM1_ESM.doc]

**QUESTIONNAIRE ON CURRENT USE OF TRYPANOCIDAL DRUGS**

(Fill in the blanks and/ or tick against the most appropriate answer)

Date of Interview...……………………….. Village……………………………………………

Ward……………………………………… District…………………………………………….

1. Identity number of respondent ………………………………………
2. Gender of respondent ;
   1. Male
   2. Female
3. Age of respondent;
   1. < 20yrs
   2. 20 – 29yrs
   3. 30 – 39yrs
   4. 40 – 49yrs
   5. 50 – 59yrs
   6. 60 – 69yrs
   7. ≥70
4. Position in family
5. Head
6. Second in charge
7. Other, specify…………………………..
8. Highest level of education attained by respondent;
   1. Informal
   2. Standard seven
   3. Form four
   4. Form six
   5. Tertiary non-degree
   6. University
9. Main occupation;
   1. Livestock farming
   2. Crop farming
   3. Agropastoral
   4. Employee
   5. Business person
   6. Other, specify………………………………..
10. Which livestock are raised:
    1. Cattle
    2. Sheep
    3. Goats
    4. Other, specify………………………..
11. Number of cattle owned;
    1. <10
    2. 10 – 20
    3. 21 – 30
    4. 31 – 40
    5. 41 – 50
    6. 51 – 100
    7. > 100
12. Types of cattle owned:

Type Number

- 1. Bulls ________________
  2. Cows ________________
  3. Male calves ________________
  4. Female calves ________________

1. Have you ever encountered diseases in your farm
   1. YES
   2. NO
2. In your farm which is the most important disease(s) affecting your cattle:
   1. Trypanosomiasis
   2. East Coast Fever
   3. Babesiosis
   4. Anaplasmosis
   5. Foot and Mouth Disease
   6. Contagious Bovine Pleuropneumoniae
   7. Brucellosis
   8. Bovine Tuberculosis
   9. Worms
3. Do you normally encounter tsetse flies in your area / where your animals graze?
   1. Yes
   2. No
4. From the pictures provided (see last page), put a tick on a tsetse picture you have been encountered in your area or animal grazing areas.
5. How do you deal with tsetse fly problem?
   1. application of insecticides
   2. bush clearing
   3. target panel
   4. more than one of the methods above
6. Do you have tick problem in your area?
   1. YES
   2. NO
7. How do you prevent tick infestation on your animals:
   1. Application of acaricide
   2. burning of pasture
   3. hand removal of ticks from animal’s body
   4. application of more than one of the above methods
8. What insecticide/ acaricide do you normally use:
   1. Pyrethroids
   2. Organophosphates
   3. Organochlorines
   4. Carbamates
   5. Other specify……………….
9. In the case of trypanosomiasis, what was the most prominent clinical sign observed:
   1. Emaciation (progressive loss of body condition)
   2. Anaemia
   3. Intermittent fever
   4. Reduced productivity
   5. Swollen lymph nodes
   6. Anorexia
   7. Loss of tail switch
10. How do you control trypanosomiasis in your farm:
    1. Tsetse control
    2. Chemoprophylaxis using trypanocides
    3. Treatment when sick animal encountered
    4. Slaughtering
    5. Other, specify………………………………………
11. If you use trypanocides, where do you obtain them from:
    1. Veterinary drug shops
    2. Veterinary professionals/ Paraprofessionals
    3. Neighbours
    4. Livestock auction markets
    5. Other, specify………….
12. How do you make diagnosis in your animals:
    1. Clinical signs
    2. Laboratory investigation
    3. Call veterinarians/paravet
13. If you control / treat, which drug do you apply most:
    1. Diminazene aceturate (Berenil)
    2. Isometamidium chloride (Samorin)
    3. Imidocarb (Imizol)
    4. Buparvaquone (Butalex)
    5. OTC
    6. Other, specify…………………………………..
14. Who treats animals:
    1. Veterinary professionals/ paraprofessionals
    2. Community animal health worker
    3. Family member
    4. Neighbour
    5. Other, specify………….
15. When you control / treat animals disease with trypanocides, which route do you normally prefer:
    1. Intramuscular
    2. Intravenous
    3. Subcutaneous
    4. Oral
    5. Topical
    6. Other, specify………………………
16. What are intervals for isometamidium/ trypanocides prophylaxis:
    1. A week
    2. Every three months
    3. Every six months
    4. Other, specify………………………………..
17. Do you know “drug withdrawal period”?
    1. YES
    2. NO
18. Meat and/ or milk obtained from an animal under treatment is:
    1. Thrown away
    2. Sold for human consumption
    3. Consumed by family members
    4. Consumed by other animals
19. If you do not observe withdrawal period, what are the reasons
    1. unaware of risks
    2. to avoid economic loss through discarding of animal products
20. Overall comments on not controlling animal trypanosomiasis using trypanocide;
21. Drugs availability
22. Willingness to control
23. Unawareness
24. Lack of Veterinary services
25. Financial constrain

Name of interviewer………………………………………………

Signature.………………………………………………………

[
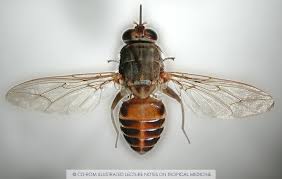
](http://www.google.co.tz/imgres?imgurl=http://itg.author-e.eu/Generated/pubx/173/mm_files/do_2716/co_67525/cd_1094_050c.jpg&imgrefurl=http://itg.author-e.eu/Generated/pubx/173/african_trypanosomiasis/vector.htm&h=481&w=756&tbnid=IqtVSsyQ_r9EzM:&docid=T1r5sObQIglzMM&ei=aEPlVbm-O7KR7AbEh4OIBA&tbm=isch&ved=0CAMQMygAMAA4ZGoVChMIuZLivpjVxwIVsgjbCh3EwwBB)[
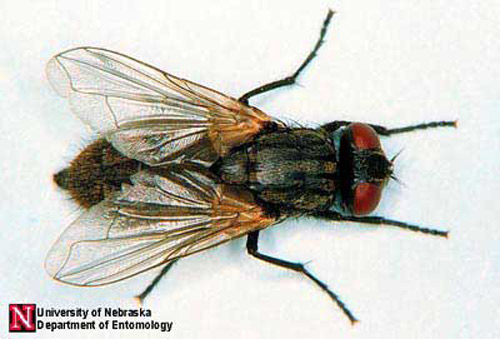
](http://www.google.co.tz/url?sa=i&rct=j&q=&esrc=s&source=images&cd=&cad=rja&uact=8&ved=0CAcQjRxqFQoTCI3j7drN2McCFUdZ2wod99MMyA&url=http://entnemdept.ufl.edu/creatures/urban/flies/house_fly.HTM&psig=AFQjCNFpBIenuOhe33gUEYkkh5nmcxqReg&ust=1441292119146196)
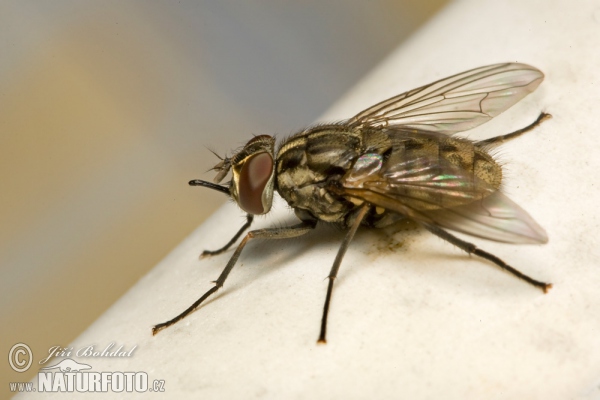
[
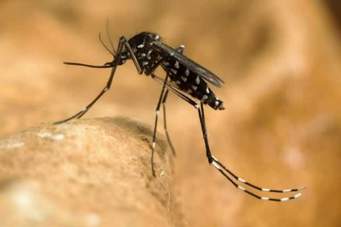
](javascript:%20void(0)) [
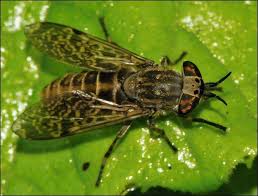
](http://diptera.info/forum/viewthread.php?thread_id=43383&pid=190156)[
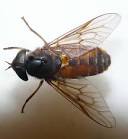
](http://www.google.co.tz/url?sa=i&rct=j&q=&esrc=s&source=images&cd=&cad=rja&uact=8&ved=0CAMQjRxqFQoTCJPVjuas2McCFTBH2wodzr8FDg&url=http://www.diptera.info/forum/viewthread.php?thread_id=50855&pid=218792&psig=AFQjCNFv-9jQ-cb6tgnK43jASFvcDApscA&ust=1441283291456013)[
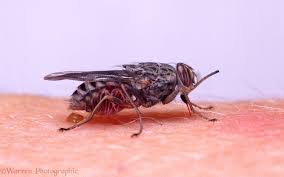
](http://www.google.co.tz/imgres?imgurl=http://www.warrenphotographic.co.uk/photography/bigs/06348-Tsetse-fly-biting-white-background.jpg&imgrefurl=http://www.warrenphotographic.co.uk/06348-tsetse-fly-biting&h=904&w=1450&tbnid=a3ONI5-QrPzUPM:&docid=P09O_dyIQY7ttM&ei=FUPlVezMCoOx7QbnsYSABQ&tbm=isch&ved=0CFoQMygyMDJqFQoTCKyr55aY1ccCFYNY2wod5xgBUA)[
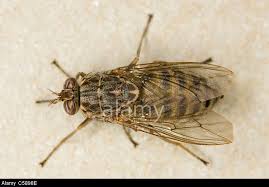
](http://www.google.co.tz/imgres?imgurl=http://c8.alamy.com/comp/C5898E/dorsal-view-of-a-female-savannah-tsetse-fly-glossina-morsitans-morsitans-C5898E.jpg&imgrefurl=http://www.alamy.com/stock-photo-dorsal-view-of-a-female-savannah-tsetse-fly-glossina-morsitans-morsitans-37676926.html&h=446&w=640&tbnid=hP15AYftLRlWCM:&docid=99868v8p987EyM&ei=G2rnVdqTEcW17gaDgK_QBg&tbm=isch&ved=0CD8QMyg8MDw4yAFqFQoTCJqNu9al2ccCFcWa2wodA8ALag)
